# A Shift in *Porcine Circovirus* 3 (PCV‐3) History Paradigm: Phylodynamic Analyses Reveal an Ancient Origin and Prolonged Undetected Circulation in the Worldwide Swine Population

**DOI:** 10.1002/advs.201901004

**Published:** 2019-09-30

**Authors:** Giovanni Franzo, Wanting He, Florencia Correa‐Fiz, Gairu Li, Matteo Legnardi, Shuo Su, Joaquim Segalés

**Affiliations:** ^1^ Department of Animal Medicine Production and Health (MAPS) University of Padua Viale, dell'Università 16 35020 Legnaro (PD) Italy; ^2^ MOE International Joint Collaborative Research Laboratory for Animal Health & Food Safety Jiangsu Engineering Laboratory of Animal Immunology Institute of Immunology College of Veterinary Medicine Nanjing Agricultural University Nanjing 210000 China; ^3^ IRTA Centre de Recerca en Sanitat Animal (CReSA, IRTA‐UAB) Campus de la Universitat Autònoma de Barcelona Bellaterra 08913 Spain; ^4^ UAB Centre de Recerca en Sanitat Animal (CReSA, IRTA‐UAB) Campus de la Universitat Autònoma de Barcelona Bellaterra 08913 Spain; ^5^ Departament de Sanitat i Anatomia Animals Facultat de Veterinària Universitat Autònoma de Barcelona Bellaterra 08913 Spain

**Keywords:** epidemiology, evolution, porcine circovirus 3 (PCV‐3), phylodynamic, phylogeography

## Abstract

The identification of a new circovirus (*Porcine circovirus* 3, PCV‐3) has raised a remarkable concern because of some analogies with *Porcine circovirus 2* (PCV‐2). Preliminary results suggest an extremely recent PCV‐3 emergence and high mutation rate. Retrospective studies prove its circulation at least since the early 1990s, revealing that PCV‐3 could have been infecting pigs for an even longer period. Therefore, a new evaluation, based on an updated collection of PCV‐3 sequences spanning more than 20 years, is performed using a phylodynamic approach. The obtained results overrule the previous PCV‐3 history concept, indicating an ancient origin. These evidences are associated with an evolutionary rate far lower (10^−5^–10^−6^ substitution/site/year) than the PCV‐2 one. Accordingly, the action of selective pressures on PCV‐3 open reading frames (ORFs) seems to be remarkably lower compared to those acting on PCV‐2, suggesting either a reduced PCV‐3 plasticity or a less efficient host‐induced natural selection. A complex and not‐directional viral flow network is evidenced through phylogeographic analysis, indicating a long lasting circulation rather than a recent emergence followed by spreading. Being recent emergence has been ruled out, efforts should be devoted to understand whether its recent discovery is simply due to improved detection capabilities or to the breaking of a previous equilibrium.

## Graphical abstract

**The results of this study overrule the previous PCV‐3 history concept,** indicating an ancient origin (in the order of several centuries or millennia) and a lower evolutionary rate (10^−5^–10^−6^ substitution/site/year) than the PCV‐2 one. A complex and not‐directional viral flow network is evidenced through phylogeographic analysis, indicating a long lasting circulation rather than a recent emergence followed by spreading.

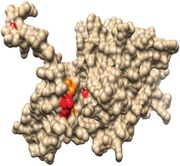

## 1 Introduction

The *Circovirus* genus claimed veterinarian attention shortly after its discovery, since its members were considered responsible of relevant diseases in birds.^1^, ^2^, ^3^ However, it was only in the middle 1990s, when *Porcine circovirus 2* (PCV‐2) and its related clinically and economically relevant syndromes (thereafter named Porcine circovirus diseases; PCVD) were recognized, that this genus became the focus of an intensive research activity.^4^, ^5^

Since then, a remarkable collection of data and knowledge has been gathered on PCV‐2 biology, pathogenesis, epidemiology, control, and evolution.^6^, ^7^ One of the most astonishing findings was the evidence that, in spite of the sudden PCVD emergence, PCV‐2 has shared a long path with domestic and wild swine populations.^8^, ^9^ Not only this but other swine infection scenarios have pointed out the role of the modern farming system in the rising of new multifactorial diseases.^4^, ^10^

The history seems to repeat itself for a new, recently discovered porcine circovirus: *Porcine circovirus 3* (PCV‐3).^11^ This virus is featured by a circular ssDNA genome of ≈2000 bases containing three open reading frames (ORFs) identified so far,^11^ although only ORF1 and ORF2 have been characterized. ORF1, located on the positive strand, apparently codes for a single replicase protein of 296–297 aa.^11^, ^12^ ORF2 is located on the negative DNA viral strand and encodes the caspsid protein.^12^ Despite the common genomic organization, PCV‐3 is distantly related to other known circoviruses, although a certain relation with bat and avian circoviruses has been suggested based on phylogenetics, codon bias and genome composition analysis.^13^, ^14^

PCV‐3 was first identified in the USA in 2015 using a metagenomics approach in tissues from animals displaying porcine dermatitis and nephropathy syndrome (PDNS) and reproductive disorders.^11^ Thereafter, it has been identified all over the world, including Asia,^15^, ^16^, ^17^, ^18^ Europe^19^, ^20^, ^21^, ^22^ and South America,^11^, ^23^ in presence of several clinical syndromes like PDNS,^11^ reproductive disorders,^24^, ^25^ respiratory disease,^26^, ^27^ and myocarditis.^28^ Moreover, PCV‐3 genome has been detected through in situ *hybridization* and immunohistochemistry in different tissue lesions,^11^, ^28^, ^29^ supporting the potential etiological role of PCV‐3. However, its identification with comparable prevalence both in healthy pigs^30^, ^31^ and wild boar^32^, ^33^, ^34^ questions the pathogenic role of this virus or at least suggests the need of other concomitant factors to trigger overt disease.

Nevertheless, based on the “PCV‐2 experience,” a pressing concern has been directed toward the study of the evolution and origin of PCV‐3: is PCV‐3 a newly originated viral species or is it an ancient one that has been circulating for a long time, only recently emerging as a potential threat for swine industry?

The first attempts to answer this question, based on a limited number of samples collected over a short time period, pointed out a recent PCV‐3 origin (approximately in the new millennium) coupled with a noteworthy evolutionary rate.^35^ However, retrospective studies performed in Sweden^20^ in 1993 and Spain^36^ and China^15^ in 1996 demonstrated that PCV‐3 has been circulating during several decades in domestic pigs. Remarkably, PCV‐3 has been detected in the oldest samples so far tested in these studies, showing a marked limit in our knowledge due to scarce data availability and suggesting that this virus could have infected pigs for even a longer period.^12^

Because of the relevance in terms of PCV‐3 epidemiology understanding and control strategies development, the present study attempts to re‐evaluate PCV‐3 history, population dynamics and spreading patterns based on a wider sequence dataset, spanning the broader collection time window currently available. The herein reported results throw a new light on PCV‐3 history and its evolutionary pathways.

## 2 Results

### 2.1 Dataset

A total of 187 complete genome sequences were included in the *dataset1*, 208 in the *dataset2* and 421 (427 nucleotide long, spanning the region between nucleotides 1347 and 1803 based on the reference genome KT869077.1) in the *dataset3*. All databases comprised sequences originating from 14 countries over a time period between 1996 and 2018, although with different number per country (Datasets S1–S3, Supporting Information).

### 2.2 PCV‐3 Origin, Evolutionary Rate, and Population Dynamics

**Figure 1:**
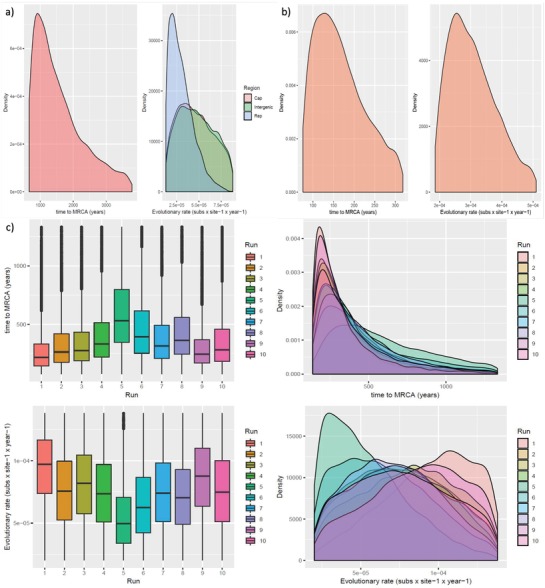
a) PCV‐3 tMRCA and evolutionary rate based on *dataset1*. Left figure: density plot of the MRCA posterior probability. Right figure: density plot of the mean evolutionary rate posterior probability. Evolutionary rates of different genomic regions have been color coded. The 95HPD intervals are reported for both figures. b) PCV‐3 tMRCA and evolutionary rate based on *dataset3*. Left figure: density plot of the MRCA posterior probability. Right figure: density plot of the mean evolutionary rate posterior probability. The 95HPD intervals are reported for both figures. c) PCV‐3 tMRCA and evolutionary rate based on *dataset2*. Upper figure: box plot (left) and density plot (right) of the MRCA posterior probability. Lower figure: box plot (left) and density plot (right) of the mean evolutionary rate posterior probability. Results have been estimated performing ten independent runs based on randomly sampled sequences. The 95HPD intervals are reported for both figures.

The analysis performed on the *dataset1* provided a time to most recent common ancestor (tMRCA) of 1372.23 years before present (ybp), although with a high uncertainness [95 high posterior density [95HPD]: 666.30–3780.91] (**Figure** **1**a and Figure S1, Supporting Information). The lower tMRCA estimate was provided by dataset3 (156.9 ybp [95HPD: 75.72–318.28]) (Figure 1b and Figure S2, Supporting Information). *Dataset2* provided an intermediate tMRCA estimation (≈300 ybp) when the results of the ten independent runs were averaged. However, a substantial overlap in the confidence intervals compared to *dataset1* was observed [95HPD: 138.79–1333.4]. The different runs, performed on 10 randomly generated sequence datasets provided consistent results (Figure 1c and Figure S3, Supporting Information).

Molecular clock estimates for the complete genome dataset reported 2.35 × 10^−5^ [95HPD: 8.79 × 10^−6^–4.71 × 10^−5^], 4.71 × 10^−5^ [95HPD: 1.75 × 10^−5^–9.38 × 10^−5^], and 5.13 × 10^−5^ [95HPD: 1.32 × 10^−5^–1.92 × 10^−5^] substitution/site/year for the ORF1, ORF2, and intergenic regions, respectively (Figure 1a).

Accordingly, the *dataset2* estimates showed an evolutionary rate in the range 10^−4^–10^−5^ substitution/site/year, consistent among different runs (Figure 1c). Finally, the evolutionary rate estimated using the *dataset3* was 2.88 × 10^−4^ [95HPD: 1.85 × 10^−4^–4.41 × 10^−4^] (Figure 1b).

**Figure 2:**
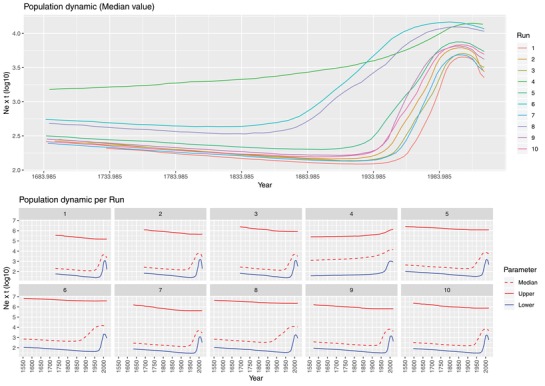
PCV‐3 genotype population dynamics reconstructed based on *dataset2*. Upper figure: mean relative genetic diversity (N e x t) of the worldwide PCV‐3 population overtime. The results of the ten independent runs have been color coded. Lower figure: median and upper and lower 95HPD values are reported for each run.

The relative genetic diversity (Ne × t) was featured by a broad 95HPD, independently from the considered dataset or run. Nevertheless, a trend toward a rise in the viral population size was observed, peaking approximately in the 1980s (**Figure** **2**).

### 2.3 Phylogeographic Analysis

**Figure 3:**
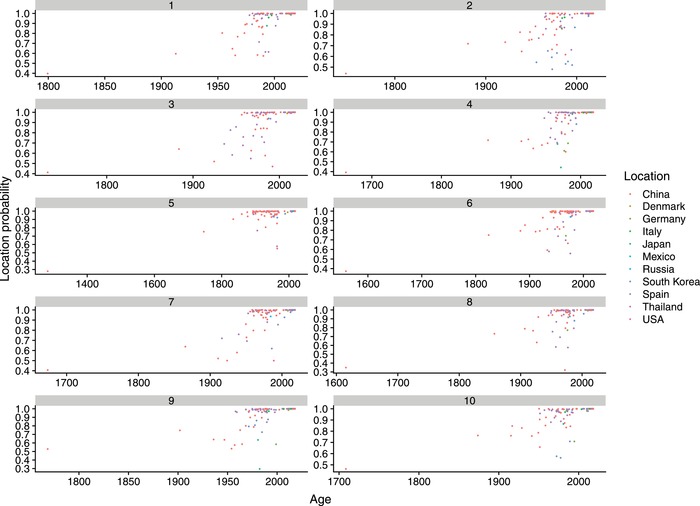
Ancestral location scatter plot. Scatter plot representing the posterior probability of each ancestral location (color coded) prediction over time. The results of ten independent BEAST run are reported.

Reconstruction of the viral spread over time demonstrated a relevant uncertainness (i.e., posterior probability lower than 0.9) in the ancestral country estimation (**Figure** **3**). Therefore, accounting for this uncertainness, relationship among strains were estimated in terms of well‐supported (i.e., Bayesian Factor (BF) > 10) contacts among countries.

**Figure 4:**
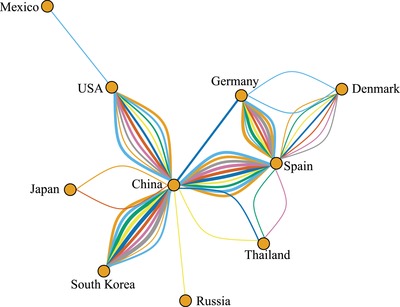
Network reporting the well‐supported migration routes (BF > 10). PCV‐3 spreading path among different countries estimated using ten independent BEAST runs (color coded) based on *dataset2*. The arrows' size is proportional to the BF value.

Except for a certain interdataset variability, the overall picture supports the presence of three interconnected nuclei of viral spread, corresponding to Asia, Europe, and America, with Asia acting as an intermediary between Europe and America (**Figure** **4** and Figures S4 and S5, Supporting Information). Within those main areas, several local migration routes were proven significant, being China the most likely responsible for viral spread to other Asian countries (e.g., South Korea, Thailand, Japan, and Russia). A more complex and less directional network could be inferred for European countries. Viral spread appears also to occur between USA and Mexico. However, the contact with the South American country included in the study (i.e., Brazil) was more likely mediated by viral strain exchange with China rather than other American countries.

### 2.4 Selective Pressure Analysis

**Figure 5:**
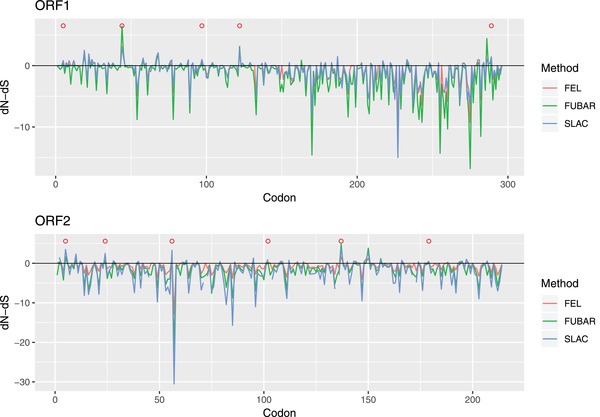
Line graph reporting dN–dS values estimated for each codon position of ORF1 (upper figure) and ORF2 gene (lower figure) using different methods. Sites detected to be under statistically significant positive selection by MEME are reported as red circles.

Selective pressure analysis demonstrated a clear dominance of sites under negative pressure both in the Rep and Cap proteins (**Figure** **5**). Limited evidences were detected of pervasive diversifying selection in both proteins: positions 19, 44, 45, and 122 of the Rep protein were proven statistically significant with the Fast Unconstrained Bayesian AppRoximation (FUBAR) method only, while position 5 of the Cap was detected by fixed effects likelihood (FEL) and FUBAR, and sites 56 and 137 by the latter method only.

On the other hand, episodic diversifying selection was detected in positions 5, 44, and 97 in the Rep, and in positions 5, 56, and 102 in the Cap proteins.

A comparison of the selective pressures acting on the two genes showed a statistically significant different selective regimen (i.e., different selection strength and proportion of sites under selection) acting on both genes (*p* = 0.003), being the overall dN/dS and proportion of sites higher in the ORF2 alignment.

**Figure 6:**
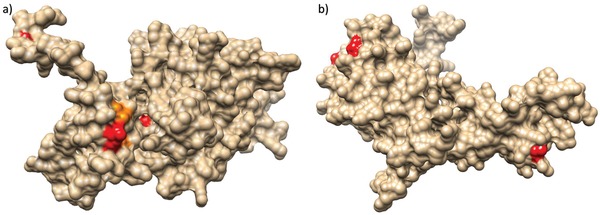
a) Tertiary structure of the Rep protein estimated through homology model. When located on the protein surfaces, sites under pervasive and episodic diversifying selection have been highlighted in orange and red, respectively. b) Tertiary structure of the Cap protein estimated through homology model. When located on the protein surfaces, sites under pervasive and episodic diversifying selection have been highlighted in red.

Homology modeling allowed an approximate reconstruction of the protein tertiary structure. Two sites (i.e., 5 and 44) under episodic diversifying selection were located on the Rep protein surface, as well as positions 19 and 45 (detected by FUBAR) (**Figure** **6**a). The remaining sites under diversifying selection were buried in the protein predicted structure. In the Cap protein, all the amino‐acids under episodic diversifying selection were located on the capsid surface (Figure 6b), while sites detected by FUBAR were located inside the protein structure.

## 3 Discussion

The identification of PCV‐3, a new porcine circovirus resembling the significant pig pathogen PCV‐2 from several perspectives, has raised a great interest toward the epidemiology and biology of this virus. One of the most relevant questions to solve is about its origin and evolution. The number of recognized swine viruses has remarkably increased in the last years^10^, ^12^ raising a dichotomous question to this phenomenon: have these viruses been recently introduced in a new host species?, or have they circulated for a long period, undetected in the domestic pig population and emerged only recently as a major threat because of concomitant factors or improved detection and research technologies? While the second explanation appears more likely for several pathogens, including PCV‐2,^4^, ^9^ their natural prior‐to‐emergence history remains largely unknown.

Predictably, similar questions arose on the PCV‐3 origin and some studies have meritoriously attempted to investigate this issue.^35^, ^37^ Preliminary results suggested a recent PCV‐3 emergence, which was located approximately at the beginning of the new millennium. However, all the mentioned studies included only sequences of PCV‐3 strains collected over a short time frame; i.e., after 2015. Therefore, a poor precision on tMRCA estimation could be forecasted, especially considering the low resolution in the collection date annotation (i.e., collection year resolution). In fact, tMRCA underestimation can also severely bias the substitution rate estimation. A recent tMRCA implies the current genetic heterogeneity originated over a limited time period, imposing a relatively fast evolutionary rate. Accordingly, PCV‐3 was reported to be the fasted evolving circovirus,^35^ which appears suspicious considering the limited genetic variability reported so far. The detection of PCV‐3 in retrospective samples collected during the 1990s in several countries from Europe and Asia confirmed its underestimated origin and claimed further analysis based on an updated genetic information availability.^15^, ^20^, ^36^

Remarkably, although in Fu et al., estimation of the “PCV‐3 only clade” still showed a recent origin, they were able to anticipate PCV‐3 emergence in the middle of the previous century by including the PorkNW2/USA/2009 strain in the coalescent analysis.^37^ This strain is genetically similar to PCV‐3 but shows a lower genome size and, although it could be reasonably classified as a defective PCV‐3 or a replicative intermediate,^38^ its classification remains controversial, mining the reliability of the results. Substantially comparable results were obtained by Saraiva et al.;^38^ however, the PCV‐3 strains included in this report were collected after 2015 and only Asia and America were represented.

Based on these premises, the present study aimed to reconstruct the evolution history and population dynamics of PCV‐3 based on a high quality and updated sequence dataset spanning a sampling time longer than 20 years (1996–2018). Despite our efforts, the currently available data are still limited and potentially biased by the different diagnostic and sequencing activities performed in different countries. Therefore, particular care was dedicated to evaluate result consistency by analyzing different genome regions and randomly generated, down‐sampled, datasets with different features (i.e., alignment length, number of sequences, presence of complete coding regions, etc.).^39^ Independently from the considered dataset, PCV‐3 origin was always backdated before 1900 at least. However, with the *dataset3* being the only exception, a far more ancient origin was supported, in the order of several centuries (*dataset2*) or millennia (*dataset1*). The large overlapping in 95HPD between the two datasets and analysis runs, further support the consistency of the results. The trend toward an increase in tMRCA with increasing sequence length supports the usefulness of adding informative sites to improve parameter estimation accuracy.^40^ Particularly, the full genome dataset displays some features that could have additionally contributed to tMRCA back‐estimate. The inclusion and independent modeling of more conserved regions, like the ORF1, could have allowed the reconstruction of more ancient events. Moreover, the use of the protein coding region allowed to implement a model that, although it cannot be considered an actual codon model (accounting for differential synonymous and nonsynonyms substitution rates), depicted the heterogeneous substitution rates among different alignment regions and codon positions in a more effective way. Several studies have pointed out the underestimation of the origin of rapidly evolving viruses and the occurrence of a “time‐dependent rate phenomenon,” where viral evolutionary rates appear to vary over time, continuously decreasing along with the timescale of rate measurement.^41^, ^42^, ^43^ Among the possible causes of these phenomena, substitution saturation, poor modeling of natural selection and inability to deal with the vast majority of substitutions occurring multiple times at a limited subset of sites (i.e., high rate heterogeneity among sites), have been advocated.^41^, ^42^, ^43^, ^44^ Therefore, because of the higher number of informative sites and more realistic model, the complete genome‐based estimations can likely be considered a more reliable tMRCA approximation. However, a potential underestimation of PCV‐3 tMRCA can still not be excluded based on its high diversity compared to other known circoviruses, stressing the need for further improvements in our mathematical modeling capabilities.

Apart from these considerations, which are far beyond the scope of the study, the achieved results consistently demonstrated that PCV‐3 origin should have occurred centuries ago. This scenario is further supported by the worldwide distribution pattern of the virus, featured by strain collected in different countries widely interspersed in the phylogenetic tree (Figures S1–S3, Supporting Information), similarly to what has previously been reported by other authors.^12^, ^38^, ^45^, ^46^

The root and ancestral node location posterior probability was often low (as indicated in Figure 3), revealing a largely expected uncertainty considering the large time frame between the estimated tMRCA and the oldest available sequences. Long branches and the lack of historical data hinder the inference of the spatial history of older viral lineages with confidence. Additionally, the long branch length is likely to conceal additional spatial movements between multiple locations.^47^ Consequently, migration patterns were evaluated in terms of ‘contact' among countries, minimizing the risk of over‐interpretation of their timing and directionality. Altogether, three major nuclei of local transmission (i.e., North America, Europe and Asia), connected by long distance transmission events were consistently identified. Such uncontrolled viral circulation can be easily explained by the recent PCV‐3 identification and by its frequent detection in healthy animals. The strain distribution along the tree and the best fitting of a symmetric migration model over the asymmetric one, poses in favor of a long lasting viral circulation rather than a recent emergence followed by progressive introduction in different countries. Nevertheless, based on the network structure and following a parsimony criterion, China seems to have played a pivotal role in the spreading of PCV‐3 both within and between continents, which could seem surprising being China a minor exporter of live swine. Despite our attempt to limit the effect of uneven sequence availability from different countries by down‐sampling the original dataset and creating randomly generated ones, a certain bias in spreading pattern inference due to the more intense sequencing activity in China cannot be totally excluded. However, fully comparable long and short range spreading pattern has already been described for other livestock pathogens, like PCV‐2 and *Infectious bronchitis virus* (IBV),^9^, ^48^, ^49^ supporting the plausibility of a comparable scenario for PCV‐3. Therefore, the actual presence of some preferential livestock‐virus “highways” can be suggested and further efforts should be dedicated to highlight the underlying causes, investigating the factors affecting viral dispersal and introducing effective control strategies, if necessary. While our hypothesis followed a parsimony criterion, other patterns could represent a more accurate depiction of PCV‐3 spreading. However, the current lack of accurate data in pig flow from most of the countries considered in the present study (especially in the time period when initial PCV‐3 dispersal likely occurred) prevents additional investigations of an association between swine or swine products trades and viral spread.

Despite the long lasting PCV‐3 circulation in the swine population, an increase in the viral relative genetic diversity (Ne x t; i.e., a proxy of population size dynamics) was observed in the last decades using a skyline plot, similarly to what previously described for PCV‐2.^9^ Common epidemiological causes could thus be hypothesized, like the alteration in the long lasting equilibrium due to pig raising conditions in the contest of expanding intensive farming.^4^ The estimated increase in viral population size, largely anticipate the identification of PCV‐3 in animals showing clinical signs. Therefore, the answer to whether the detected rise mirrors an increased pathogenic role of PCV‐3 or is simply due to a wider viral circulation (of no clinical significance) in a bigger and more connected animal population world, remains elusive. Although some evidences appear to support a certain association between PCV‐3 and clinical disease, contradictory reports have been published up to date and more extensive studies should be performed.

The ancient origin of PCV‐3 implies also a lower evolutionary rate compared to PCV‐2 and previous PCV‐3 estimation, both reporting a substitution rate in the order of magnitude of 10^−3^ substitution/site/year.^9^, ^35^, ^50^ On the contrary, the present study results showed a far lower rate (i.e., ≈10^−5^ substitution/site/year), which is consistent with the limited genetic variability so far observed and with the high similarity between recent sequences and those obtained from early‐mid 1990s.^12^ This evidence could suggest a lower intensity of diversifying selective pressures shaping the evolution of this virus.

Accordingly, compared with previous studies evaluating the forces acting on PCV‐2, the number of sites under positive selection in the Cap was remarkably lower in PCV‐3.^9^, ^51^ Although further confirmation will be needed, this scenario could be due to a lower plasticity of PCV‐3 or to a less intense host‐induced natural selection, which could be tentatively considered as an evidence of a lower PCV‐3 virulence and/or prolonged virus‐host co‐evolution, leading to a decreased immune response stimulation. Unfortunately, no reliable experimental in vitro and/or in vivo model is currently available to investigate the PCV‐3 immunopathogenesis and interaction within the host, which are likely the most relevant determinants of its evolution. Overall, the field of PCV‐3 immunology is still in its infancy and further confirmation must be provided to shed light on this hypothesis plausibility. Nevertheless, the phylodynamic approach implemented in the present study, based on a global viral sampling spanning more than 20 years, can provide a useful and consistent depiction of the overall patterns and determinants of PCV‐3 evolution, avoiding assumptions and constraints of viral biology induced by experimental conditions.

In fact, the ORF1 gene demonstrated a tendency toward a lower substitution rate compared to the ORF2, which was reflected by a statistically significant difference in diversifying selection acting on the two coding regions. These results, coupled with the location on the protein surface of sites under diversifying selection, suggest at least a limited action of the host immune response in shaping PCV‐3 evolution. However, it must be stressed that the homology based estimation of Rep and Cap protein conformation should be evaluated with caution because of the absence of closely related experimentally derived tertiary structures.

Overall, the present study provides an updated representation of PCV‐3 origin, population dynamics and evolution, pointing out a quite ancient viral origin and a low evolutionary rate compared to other circoviruses of clinical relevance. These results could contribute to the evaluation of the actual PCV‐3 relevance for swine industry and, possibly, to the planning of effective control strategies. It must be emphasized that the present work just scratched the surface of PCV‐3 history and biology and future and constant re‐evaluation of the present results will be mandatory to update and improve the knowledge of this emergent virus behavior.

## 4 Experimental Section

*Dataset Preparation*: All currently available PCV‐3 sequences were downloaded from Genbank (accessed on 2018 November 29) and annotated with collection year and country when available. Sequences lacking of these data were removed from the dataset. Despite the fact that the obtained sequence collection was the broader currently available, the molecular epidemiology information was still limited and sparse.

To deal with unbalanced sequence availability, which could bias the results, different sequence datasets were prepared to alternatively benefit from the higher sequence length or number (i.e., representativeness): *dataset1* included all available complete genome; *dataset2* comprised long PCV‐3 sequences (1000bp), including the Spanish ones obtained during a retrospective study conducted from the mid‐1990s onward;^36^ *dataset3* was based on a region where the higher number of PCV‐3 sequence had a full coverage.

Each dataset was designed using the following approach:

*Dataset1*: Complete genome sequences were divided in ORF1, ORF2, and intergenic regions (both intergenic regions were merged in a single partition). Coding regions were aligned at amino‐acid level and then back translated to nucleotide sequence using the MAFFT algorithm^52^ implemented in TranslatorX.^53^ Poorly aligned sequences as well as those showing premature stop codons or frame‐shift mutation were excluded from the analysis. Recombination analysis was performed using RDP4^54^ on complete genome alignment and each ORF independently.

*Dataset2*: This dataset was primarily designed to benefit from all the 1990s samples, including the partial ones describe by Klaumann et al.^36^ Additionally, *dataset2* was used to evaluate the impact of sampling bias in analysis results. To this purpose, ten independent datasets were generated by randomly sampling a maximum of ten sequences for each country‐year pair, as described by Franzo et al.^48^, ^49^ The obtained sequences were aligned using MAFFT version 7.271^52^ and scanned for recombination events using RDP4.^54^

*Dataset3*: All the available, partial and complete, PCV‐3 sequences were aligned using MAFFT^52^ and the alignment region with the highest sequence coverage was selected for further analysis. All sequences spanning the selected region were extracted, realigned, and scanned for recombination events using RDP4.^54^

*Population Parameters Estimation/Phylodynamic Analysis*: On each of the above‐mentioned datasets, a tip dated serial coalescent analysis was performed using the Bayesian approach implemented in BEAST 1.8.2^55^ to primarily estimate tMRCA, evolutionary rate, and population dynamics over time. Additionally, a discrete state phylogeography was performed as described by Lemey et al.^56^ Additionally, the implementation of the Bayesian stochastic search variable selection (BSSVS) allowed a BF test that identified the most parsimonious description of the spreading process. A BF > 10 was considered as suggestive of a significant migration pattern between country pairs.

For the *dataset1*, the three partitions (ORF1, ORF2, and intergenic regions) were allowed independent substitution and clock models, while a single tree topology for the three regions was constrained. Additionally, ORF1 and ORF2 regions were further partitioned allowing independent evolution models for each codon position. On the other hand, a single partition was used for *dataset2* and *dataset3*.The substitution model was selected based on the BIC scores calculated using Jmodeltest version 2.1.7. Molecular clock, population dynamics model, and discrete trait substitution model (i.e., symmetric vs asymmetric migration rate) were selected by evaluation of the BF (i.e., the ratio of the compared model marginal likelihoods, estimated using a Path sampling and Stepping stone approach) as suggested by Baele et al.^57^ Relaxed lognormal molecular clock,^58^ skyline population model,^59^ and symmetric migration rate were selected.^56^

The tree and the model parameters were estimated over a 100 million generation Markov Chain Monte Carlo (MCMC) chain, sampling them every 10 000 generations. Run results were accepted only if the mixing and convergence, visually inspected using Tracer,^60^ were adequate and the estimated sample size (ESS) was higher than 200, after discharging the first 20% generations as burn‐in. Parameter estimation were summarized as median and 95HPD. The maximum clade credibility tree was estimated using the treeannotator tool of the BEAST 1.8.2^55^ package. BF of well‐supported migration rates was calculated using SpreaD3.^61^

*Selective Pressure Analysis*: The presence of protein sites under diversifying selection was evaluated using several dN–dS based methods. To this purpose, all complete ORF1 and ORF2 sequences were downloaded from Genbank and aligned at codon level using TranslatorX.^53^ The achieved alignments were tested for pervasive diversifying selection using single‐likelihood ancestor counting (SLAC), FEL, and FUBAR^62^, ^63^ methods implemented in HyPhy.^64^ Significance level was set to *p* < 0.05 for SLAC and FEL and to a posterior probability higher than 0.9 for FUBAR. Sites were considered under pervasive diversifying selection when detected by at least two methods.

Since adaptive evolution often occurs in episodic bursts,^65^ i.e., affecting a small subset of branches at individual sites, episodic diversifying selection was also investigated using MEME.^66^ The MEME significance level was set *p* < 0.05.

The tertiary structure of PCV‐3 Rep and Cap proteins were estimated by homology modeling using Phyre 2.0^67^ to predict sites location under diversifying selection.

The action of selective pressures was also compared among different genes (ORF1 and ORF2) using the dNdSDistributionComparison.bf implemented in HyPhy.^64^ Particularly, the presence of a difference between the two ORFs in selective pressure strength, proportion of sites under selective pressure and selective regime (i.e. a combination of the two factors) was evaluated.

## Conflict of Interest

The authors declare no conflict of interest.

## Supporting information

Supplementary

Supplementary

Supplementary

Supplementary
