# Supplementary material for: A Shift in Porcine Circovirus 3 (PCV‐3) History Paradigm: Phylodynamic Analyses Reveal an Ancient Origin and Prolonged Undetected Circulation in the Worldwide Swine Population
Source: Adv Sci (Weinh). 2019 Sep 30;6(22):1901004. doi: 10.1002/advs.201901004 (PMC6865002; doi:10.1002/advs.201901004)
Supplement: Supplementary file 1 — Supplementary [file ADVS-6-1901004-s001.pdf]

Copyright WILEY-VCH Verlag GmbH & Co. KGaA, 69469 Weinheim, Germany, 2019.

ADVANCED SCIENCE
Open Access

Supporting Information

for *Adv. Sci.,* DOI: 10.1002/advs.201901004

A Shift in *Porcine Circovirus* 3 (PCV-3) History Paradigm: Phylodynamic Analyses Reveal an Ancient Origin and Prolonged Undetected Circulation in the Worldwide Swine Population

Giovanni Franzo, Wanting He, Florencia Correa-Fiz, Gairu Li, Matteo Legnardi, Shuo Su, and Joaquim Segalés*

Supplementary figure 1. Time calibrated phylogenetic trees based on *dataset1*. The tree branches have been color-coded according to the location predicted with the highest posterior probability.

Supplementary figure 2. Time calibrated phylogenetic trees based on *dataset3*. The tree branches have been color-coded according to the location predicted with the highest posterior probability.

Supplementary figure 3. Time calibrated phylogenetic trees based on *dataset2*. Time calibrated phylogenetic trees obtained using ten independent BEAST runs are reported. The tree branches have been color-coded according to the location predicted with the highest posterior probability.

Supplementary figure 4. Network reporting the well supported migration routes (BF>10). PCV-3 spreading path among different countries estimated using *dataset1*. The arrows' size is proportional to the BF value.

Supplementary figure 5. Network reporting the well supported migration routes (BF>10). PCV-3 spreading path among different countries estimated using *dataset3*. The arrows' size is proportional to the BF value.

Complete Genome

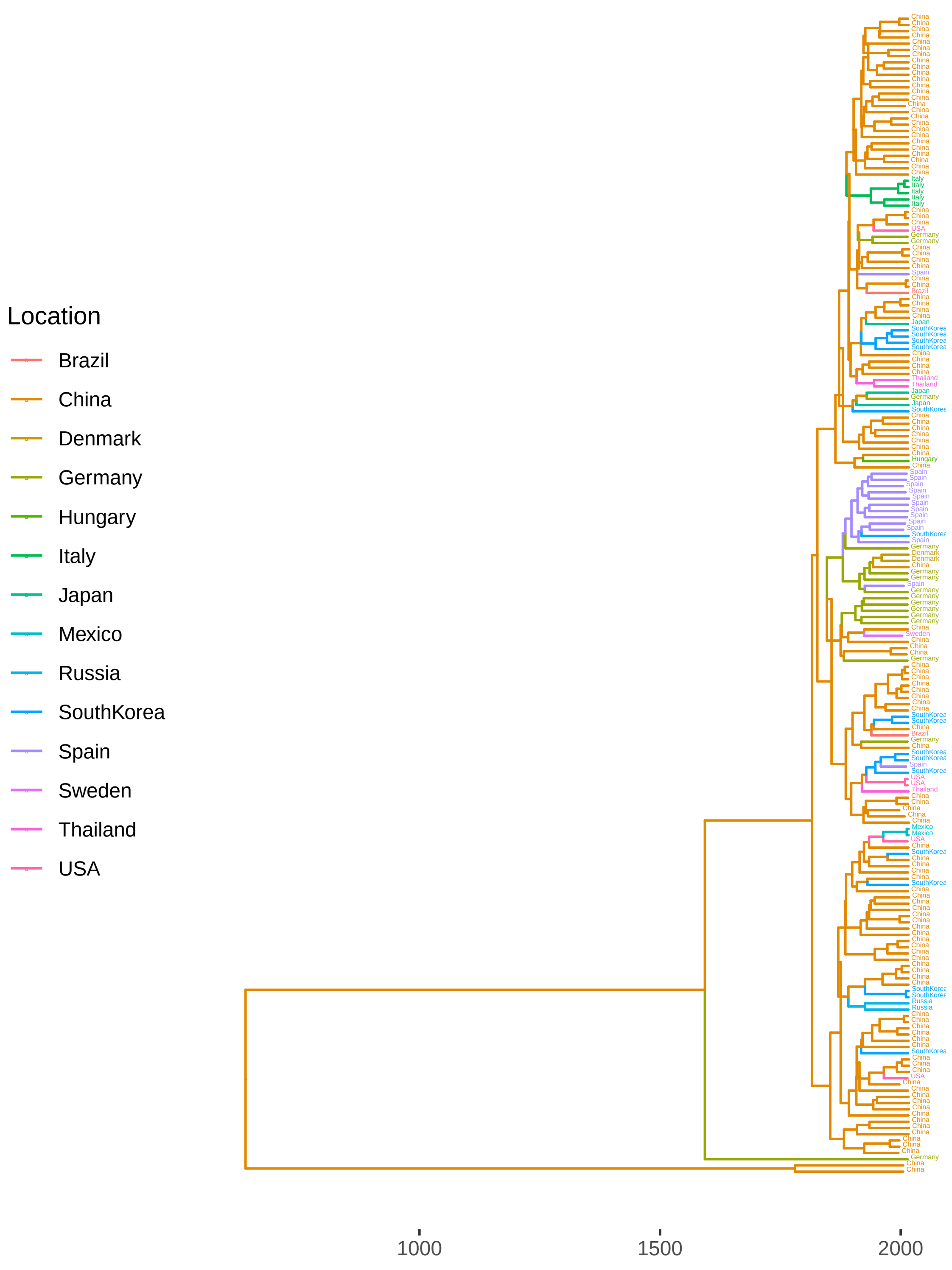

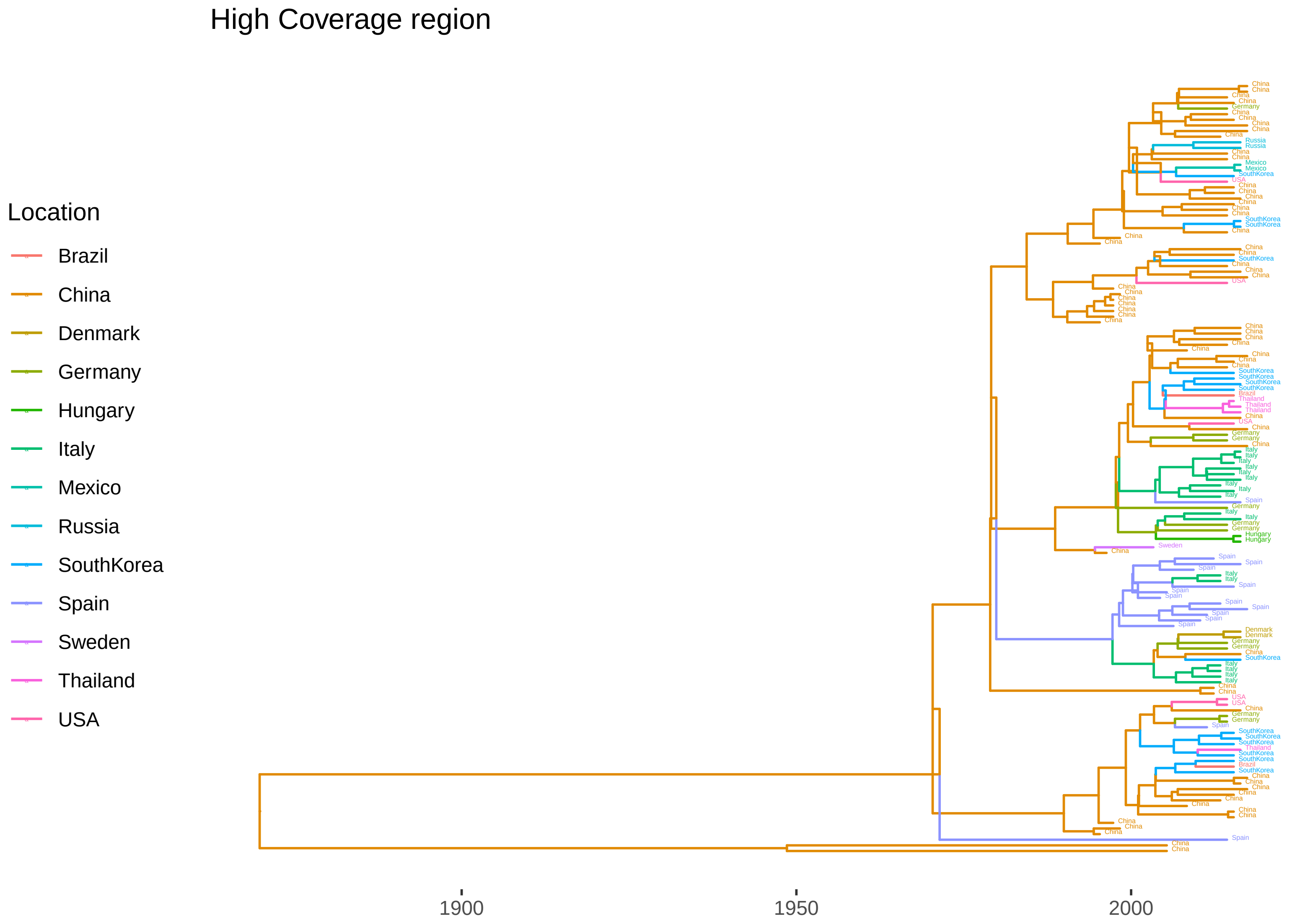
High Coverage region
Location
Brazil
China
Denmark
Germany
Hungary
Italy
Mexico
Russia
SouthKorea
Spain
Sweden
Thailand
USA
1900
1950
2000

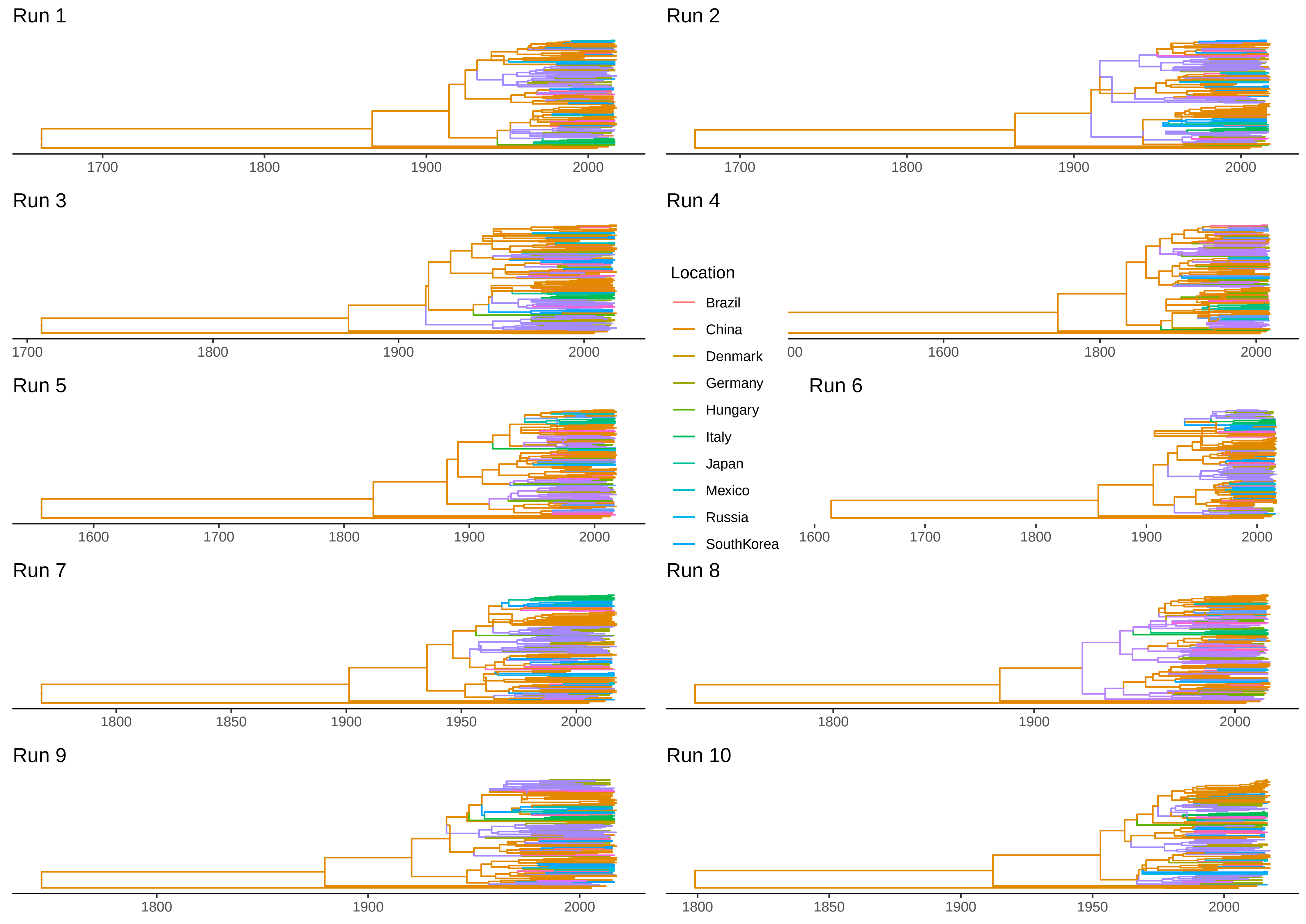
Run 1
1700
1800
1900
2000
Run 2
1700
1800
1900
2000
Run 3
1700
1800
1900
2000
Run 4
00
1600
1800
2000
Location
Brazil
China
Denmark
Germany
Hungary
Italy
Japan
Mexico
Russia
SouthKorea
Run 5
1600
1700
1800
1900
2000
Run 6
1600
1700
1800
1900
2000
Run 7
1800
1850
1900
1950
2000
Run 8
1800
1900
2000
Run 9
1800
1900
2000
Run 10
1800
1850
1900
1950
2000

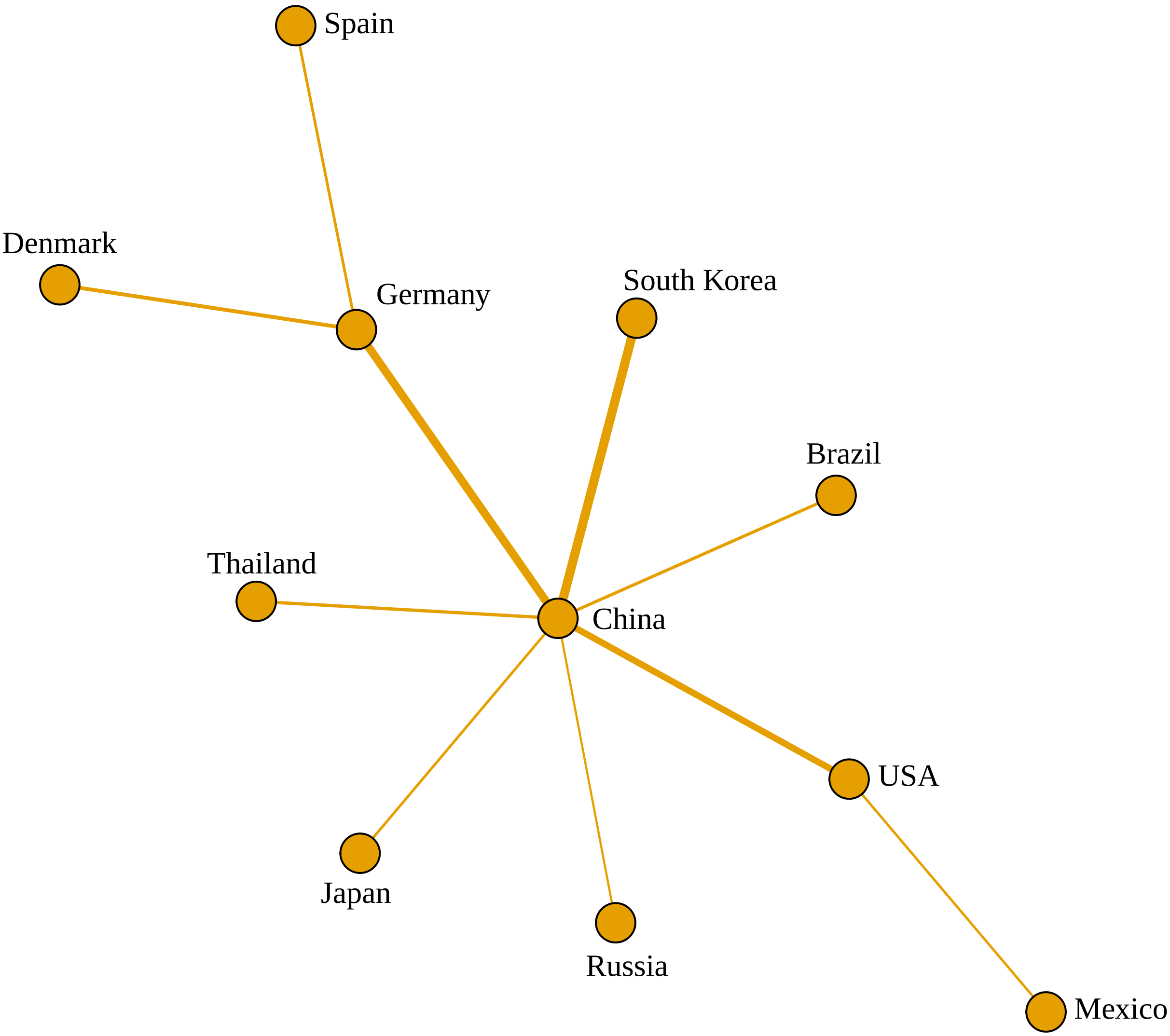
Spain
Denmark
Germany
South Korea
Brazil
Thailand
China
USA
Japan
Russia
Mexico

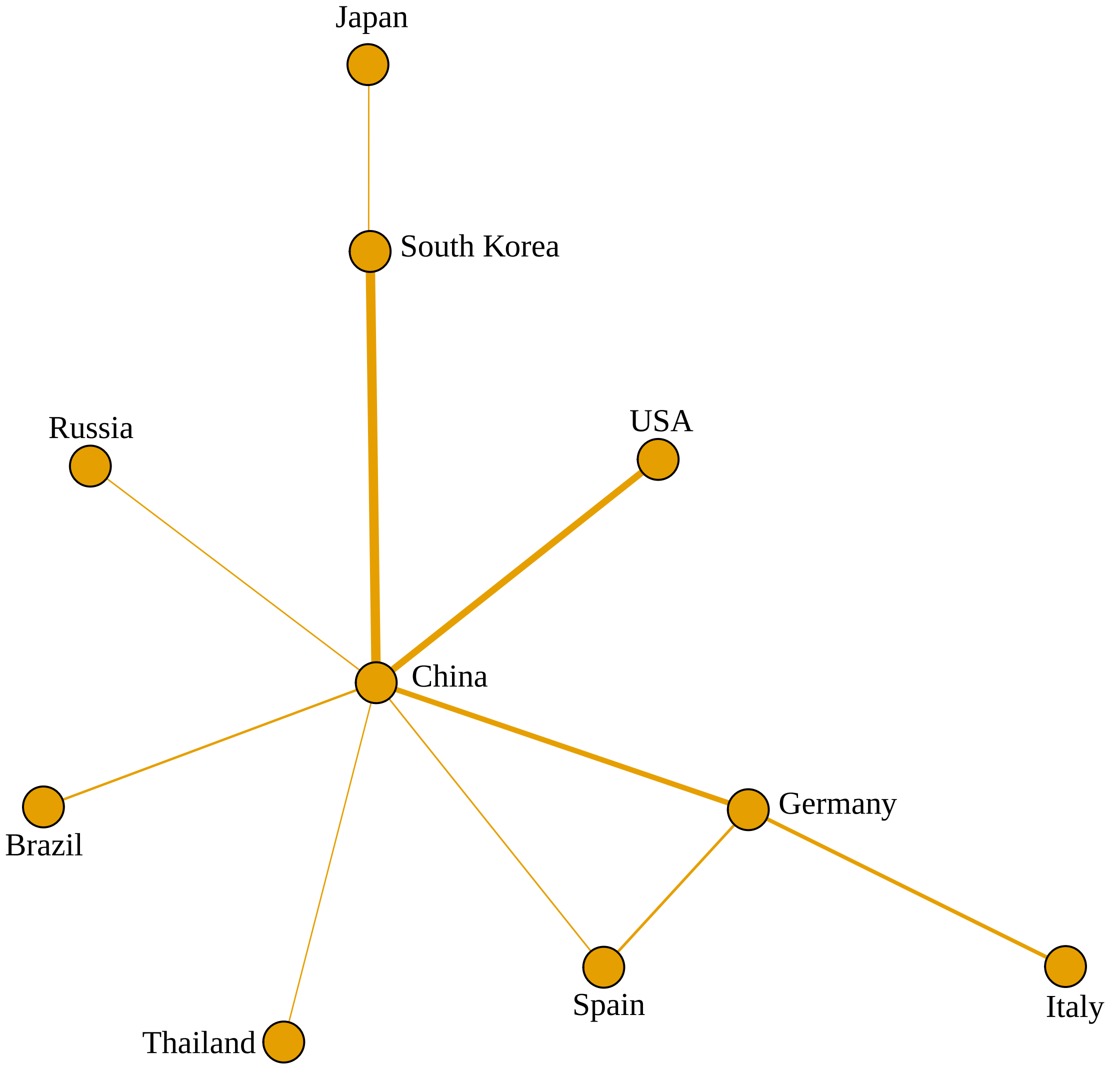
Japan
South Korea
Russia
USA
China
Germany
Brazil
Spain
Italy
Thailand
